# Attribution of changes in the trend and temporal non-uniformity of extreme precipitation events in Central Asia

**DOI:** 10.1038/s41598-021-94486-w

**Published:** 2021-07-22

**Authors:** Shan Zou, Jilili Abuduwaili, Weili Duan, Jianli Ding, Philippe De Maeyer, Tim Van De Voorde, Long Ma

**Affiliations:** 1 State Key Laboratory of Desert and Oasis Ecology, Xinjiang Institute of Ecology and Geography, Chinese Academy of Sciences, Ürümqi, 830011 China; 2 Research Centre for Ecology and Environment of Central Asia, Chinese Academy of Sciences, Ürümqi, 830011 China; 3 University of Chinese Academy of Sciences, Beijing, 100049 China; 4 College of Resource and Environment Sciences, Xinjiang University, Ürümqi, 830046 China; 5 Department of Geography, Ghent University, 9000 Ghent, Belgium; 6 Sino-Belgian Joint Laboratory of Geo-Information, 9000 Ghent, Belgium; 7 Sino-Belgian Joint Laboratory of Geo-Information, Ürümqi, 830011 China

**Keywords:** Climate sciences, Hydrology

## Abstract

Extreme precipitation events exhibit an increasing trend for both the frequency and magnitude on global and regional scales and it has already proven the impact of man-made global warming on the extreme precipitation amplification. Based on the observed datasets and global climate model (GCM) output, this study has evaluated the impact from anthropogenic forcing on the trend and temporal non-uniformity (i.e. increase in unevenness or disparity) of the precipitation amounts (PRCPTOT), extremes (R95p and RX5day) and intensity (SDII) in Central Asia (CA) from 1961 to 2005. Results indicate that radiative forcing changes, mainly driven by human activities, have significantly augmented the extreme precipitation indices in CA. The median trend with the influence of anthropogenic activities for the PRCPTOT, SDII, R95p and RX5day amounted to 2.19 mm/decade, 0.019 mm/decade, 1.39 mm/decade and 0.21 mm/decade during the study period, respectively. A statistically insignificant decrease in non-uniformity was noticed for the PRCPTOT, SDII and RX5day in Central CA (CCA) and Western CA (WCA), while Eastern CA (ECA) was the only region with a statistically significant increase in non-uniformity of the PRCPTOT, SDII, R95p and RX5day by 4.22%, 3.98%, 3.73% and 3.97%, respectively from 1961 to 2005 due to anthropogenic forcing. These results reflect the difference in various regions regarding the impact of anthropogenic forcing on the non-uniformity of extreme precipitation events in CA, which might help to fully understand the role of anthropogenic forcing in the changes of the precipitation extremes in CA and contribute to the development of water resource management strategies.

## Introduction

Extreme precipitation events have a striking impact on the natural environment and society and were put under tremendous attention due to their significant damage on the ecosystems, fatalities and economic losses. Since the 1960s, a dramatic change in extreme precipitation events has been observed on the global and regional scales and most parts of the globe have shown a rising trend in the frequency and magnitude of global warming^1^, causing more precipitation-related disasters such as floods, droughts, landslides and so on. From 2000 to 2019, for example, the statistics obtained from the Global Emergency Disaster Database (EM-DAT), illustrated that floods have accounted for 44% of all disaster events, affecting 1.6 billion people over the world, the worst loss for all disaster types and all these events caused serious environmental damage and pollution. Moreover, extreme precipitation events are expected to be further enhanced under continuous global warming for both the theoretical arguments and climate model projections (i.e. the Coupled Model Intercomparison Project phase 3 and 5 (CMIP3 and CMIP5))^2^, thus probably causing a greater effect on the aquatic and terrestrial ecosystems, human societies and the economy. Therefore, it is important to explore and understand the changes in the precipitation variability, especially for the extreme precipitation events.

The extreme precipitation amplification is a prominent phenomenon of the precipitation variability and recent studies have pointed out the influence of the natural variability and human activities on the precipitation variability. Some studies have demonstrated that global warming caused by anthropogenic forcing contributed to the occurrence of a few heavy precipitation events due to the increase of the moisture holding capacity of the atmosphere potentially under rising temperature^3,4^. The notable examples of this view (with a discernible human component) contain the Great Colorado Flood of September 2013^5^, extreme rainfall events during early July 2014 in Northland, New Zealand^6^ and the July 2016 extreme precipitation in China’s Wuhan^7^. However, a growing number of studies indicate that the mechanism alone is not sufficiently explanatory and more complex mechanisms and other factors might be involved as well in some (or many) of the recent strong or even unprecedented precipitation extremes^1,8^. All these mechanisms in climate change are not only affected by anthropogenic but also by natural forcing^9,10^. Therefore, attribution of climate change is a key aspect of the understanding of extreme precipitation risks and multiple methods, including the statistical and modeling methodology, have been developed in order to quantify and evaluate the respective influence from the natural and anthropogenic forcing^11–13^. Most extreme event attribution assessments have mainly focused on the impact of natural and anthropogenic forcing on the spatio-temporal variability of the annual (or seasonal) precipitation, based on the extreme climate indices such as the “Expert Team on Climate Change Detection and Indices” (hereafter ETCCDI)^14^ and global climate model (GCM) output (e.g. CMIP3 and CMIP5)^15^.

The evidence for the long-term effects of global climate change on the spatiotemporal precipitation characteristics in Central Asia (CA) is plentiful^16–19^ because CA has a unique ecological pattern with the coexistence of deserts, oases, mountains, snow and glaciers, which are extraordinarily sensitive to the warming temperature^20–22^. Precipitation is generally rare with limited wet season rainfall and snow in CA. In recent years, extreme precipitation events exhibited an upward trend, suggesting an increasing contribution to the total precipitation, which have greatly increased the risk of water-related disasters and affected water resources from mountains^22,23^. Many efforts have been made to evaluate the precipitation variability on different scales in CA, based on the sparse gauge precipitation data, GCM simulations and fusion remote sensing data which indicate an overall rise in the spatial diversity and heterogeneity of the precipitation extremes during past decades^24–29^ and future periods^30,31^. For these changing patterns, some authors have meanwhile investigated the possible influence from humidity, the atmospheric circulation and other natural factors more specifically^27,32^. Also, several studies have analyzed and distinguished the degree extent of natural and anthropogenic forcing and suggest that the latter plays an important role in the precipitation variability of CA^33^. However, this type of attribution research is still rare and further studies should be carried out so as to investigate and quantify the individual influence of anthropogenic climate change on the precipitation variation in CA.

Therefore, 4 indices including the total annual wet-day precipitation (PRCPTOT), the maximum 5-day precipitation amount (RX5day), the simple daily intensity index (SDII) and the extremely wet days (R95p) have been selected to evaluate the impact from anthropogenic forcing on the trend and non-uniformity (i.e. increase in unevenness or disparity) on the extreme precipitation events in CA. Based on the administrative boundaries, societal and geographical conditions and precipitation variability, we divided CA into 4 parts. Figure S1, Table S1) including Northern CA (NCA), Western CA (WCA), Central CA (CCA) and Eastern CA (ECA) in order to completely analyze the detailed changes of the extreme precipitation events. This study aims to (1) examine the spatial–temporal variation including the trend in historical precipitation, (2) investigate the human contribution to potential changes in precipitation non-uniformity, and (3) discuss potential influencing factors in the temporal precipitation variability of CA.

## Results

### Evaluation of the historical simulations

Because of the fact that the SDII, R95p and RX5day have no value in many locations in CA, we only carried out the evaluation for the RCPTOT from 1961 to 2005. Figure S2 shows the CMIP5 performance on the temporal variation of the historical simulations in CA, revealing a reasonable reproducibility in the magnitude and spatial patterns of the temporal variations. An obvious regional characteristic has also been observed and higher Gini-coefficients were mainly found in the southern parts for both the HadEX3 and CMIP5. The highest Gini-coefficient exceeded 0.25, which was detected in the Chinese Tarim River Basin.

Figure S2c–d suggest that the CMIP5 historical simulations of the PRCPTOT underestimate the Gini-coefficients in the southeastern regions of CCA (especially the southern edge of the Tarim River Basin), while overestimating the latter in the southwestern regions of WCA and the northern edge of the Tarim River Basin. The average bias in percentage [(CMIP5 − HadEX3)/HadEX3 × 100%] amounts to about 15.11% for the RCPTOT in CA (Figure S2d). Overall, although the CMIP5 historical simulations overestimated the observed Gini-coefficients, they generally captured the spatial patterns of the annually observed precipitation variability in CA and might be used to quantify the anthropogenic contribution to changes in the temporal precipitation variability.

### Trend variability of the precipitation due to anthropogenic forcing

**Figure 1:**
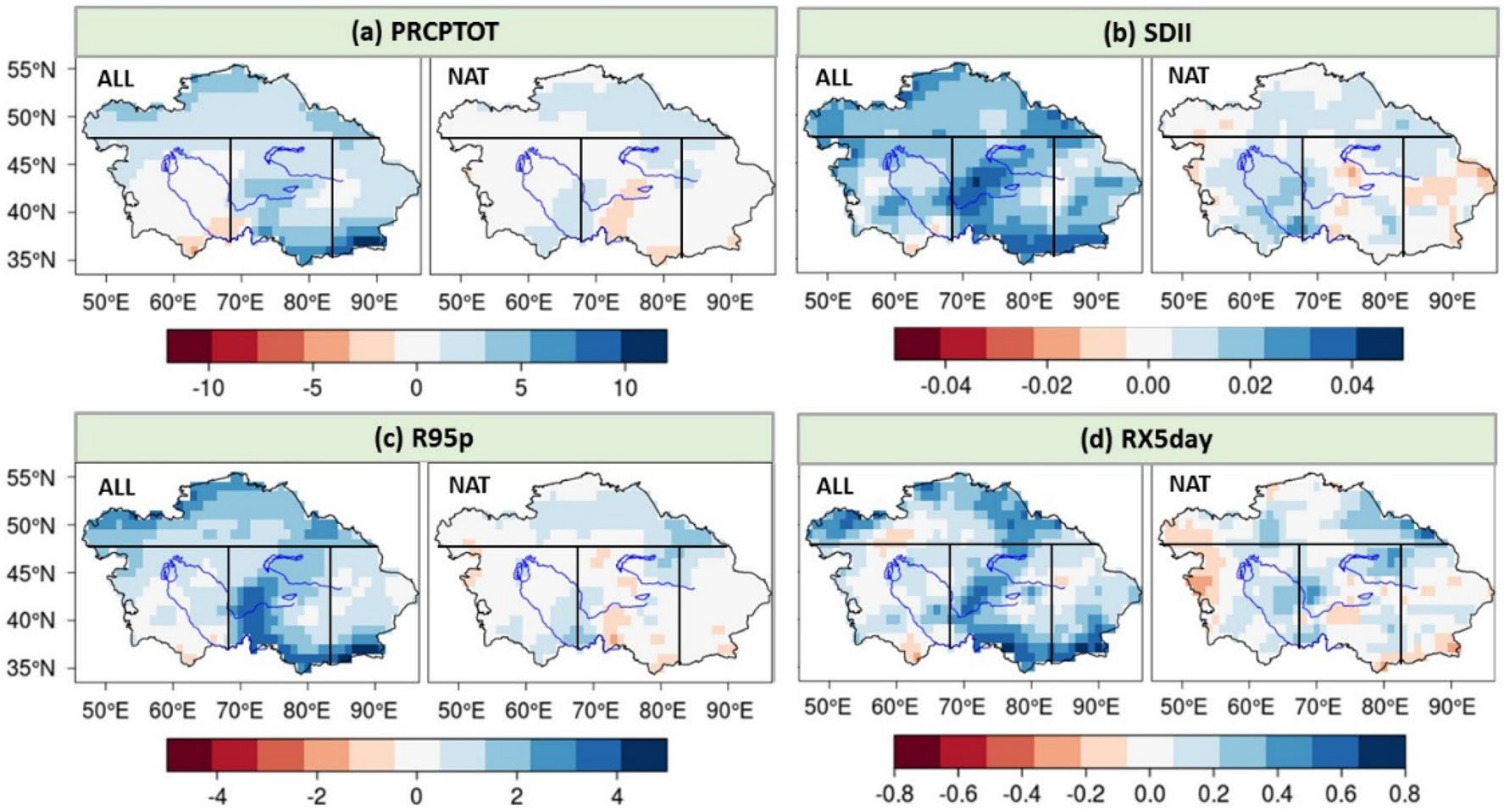
Spatial distribution of trends (ensemble means, mm/decade) for the PRCPTOT (**a**), SDII (**b**), R95p (**c**) and RX5day (**d**) under the CMIP5 ALL and NAT simulations during 1961–2005. The subfigures were done in the software R 4.0.2 (https://cran.r-project.org/bin/windows/), and then the subfigures were merged by using the Microsoft PowerPoint 2013 software (https://www.microsoft.com/).

Figure 1 shows the spatial distribution of the precipitation extreme indices’ trend under the CMIP5 ALL and NAT simulations during the period 1961–2005, indicating that ALL simulations generally have higher trends than those under the NAT simulations for all 4 indices in CA. The largest trend of the PRCPTOT measured about 10 mm/decade due to the influence of anthropogenic activities, while it amounted to approximately 3 mm/decade with no anthropogenic forcing (Fig. 1a). In terms of spatial distribution, the simulated trend of the PRCPTOT is larger in the southeastern fringe and Tianshan Mountains of CA than in other regions and we could even see a decreasing trend in the southern edge of the Aral Sea Basin due to anthropogenic forcing (Fig. 1a). A similar distribution was found for the changes of the SDII, R95p and RX5day, with the largest trend exceeding 0.04, 4.00 and 0.80 mm/decade, respectively.

**Figure 2:**
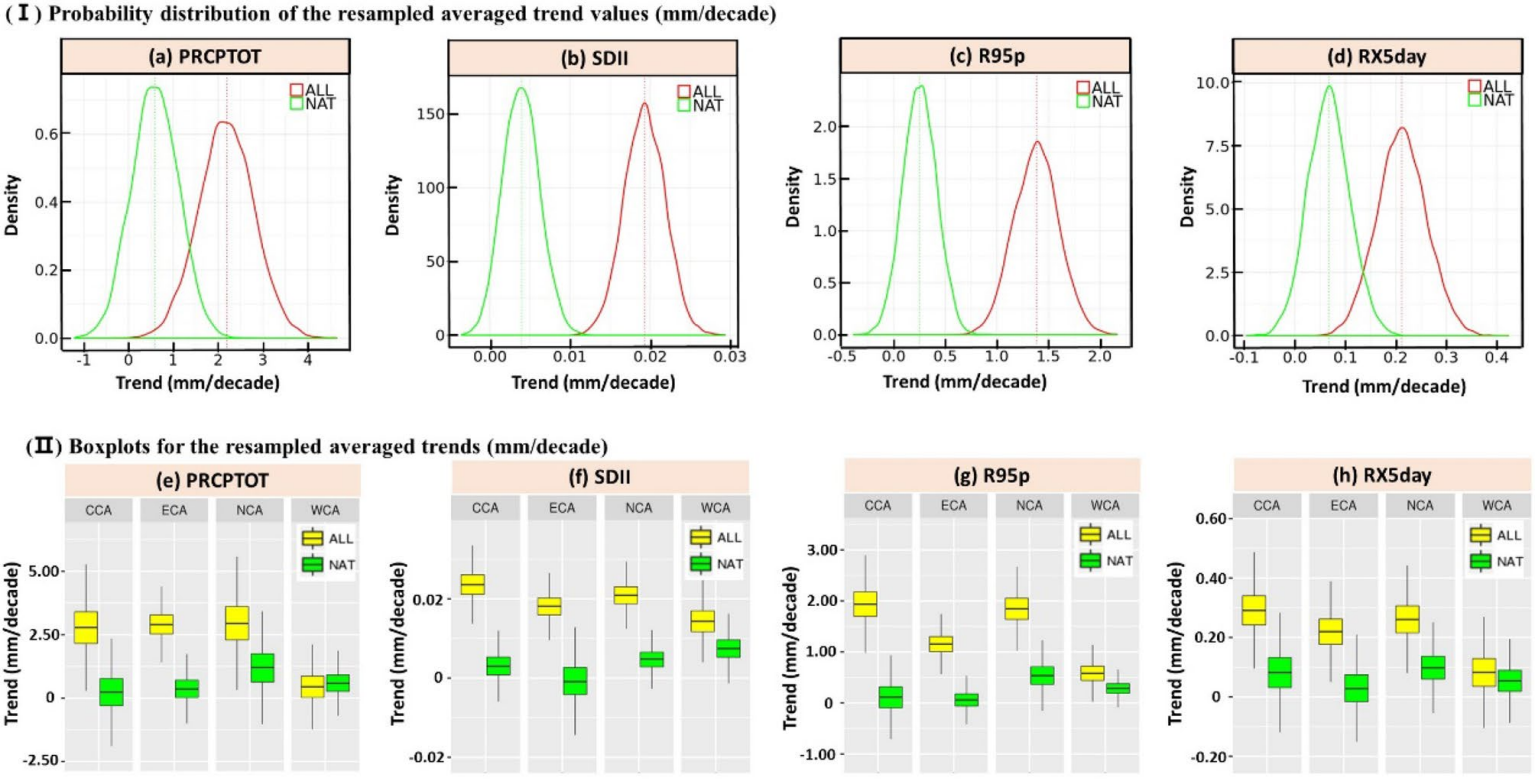
(I) Probability distribution of the resampled averaged trend values (mm/decade) for the PRCPTOT (**a**), SDII (**b**), R95p (**c**) and RX5day (**d**) under the CMIP5 ALL and NAT simulations during the period 1961–2005. The vertical line indicates the median (best estimate) trend value. (II) Boxplots for the resampled averaged trends (mm/decade) for the PRCPTOT (**e**), SDII (**f**), R95p (**g**) and RX5day (**h**) for 4 sub-regions in CA regarding the CMIP5 ALL and NAT scenario obtained by the bootstrapping procedure. The subfigures were done in the software R 4.0.2 (https://cran.r-project.org/bin/windows/), and then the subfigures were merged by using the Microsoft PowerPoint 2013 software (https://www.microsoft.com/).

Figure 2a–d show the probability distribution of the resampled average trend values for the PRCPTOT, SDII, R95p and RX5day under the CMIP5 ALL and NAT simulations during 1961–2005, which signifies that a positive shift has been noticed for all 4 indices from the CMIP5 NAT to ALL scenarios and the value of the median trend rose from 0.59 to 2.19 mm/decade, 0.004 to 0.019 mm/decade, 0.25 to 1.39 mm/decade and 0.07 to 0.21 mm/decade, respectively. These results indicate that the radiative forcing changes mainly driven by human activities are responsible for the significant rise of the extreme climate indices in CA.

Figure 2e–h illustrate the boxplots of the resampled averaged trends of 4 precipitation indices in 4 CA sub-regions under the CMIP5 ALL and NAT scenario. Except for the PRCPTOT in the WCA region, the median trend value for the ALL scenario was greater than for the NAT scenario in CCA, NCA and WCA, suggesting a human contribution to potential changes in the increase of the climate precipitation indices in CCA, NCA and WCA. Generally, CCA has the largest difference of the median averaged trends between the CMIP5 ALL and NAT scenario, which means that the largest augmentation in the trends of all 4 precipitation indices has been detected in CCA due to anthropogenic forcing. On the contrary, WCA shows the lowest values of the median averaged trends, illustrating that there are not many big changes in the PRCPTOT, SDII, R95p and RX5day under human activities in the southwestern part of CA.

### Changes in the precipitation non-uniformity due to anthropogenic forcing

**Figure 3:**
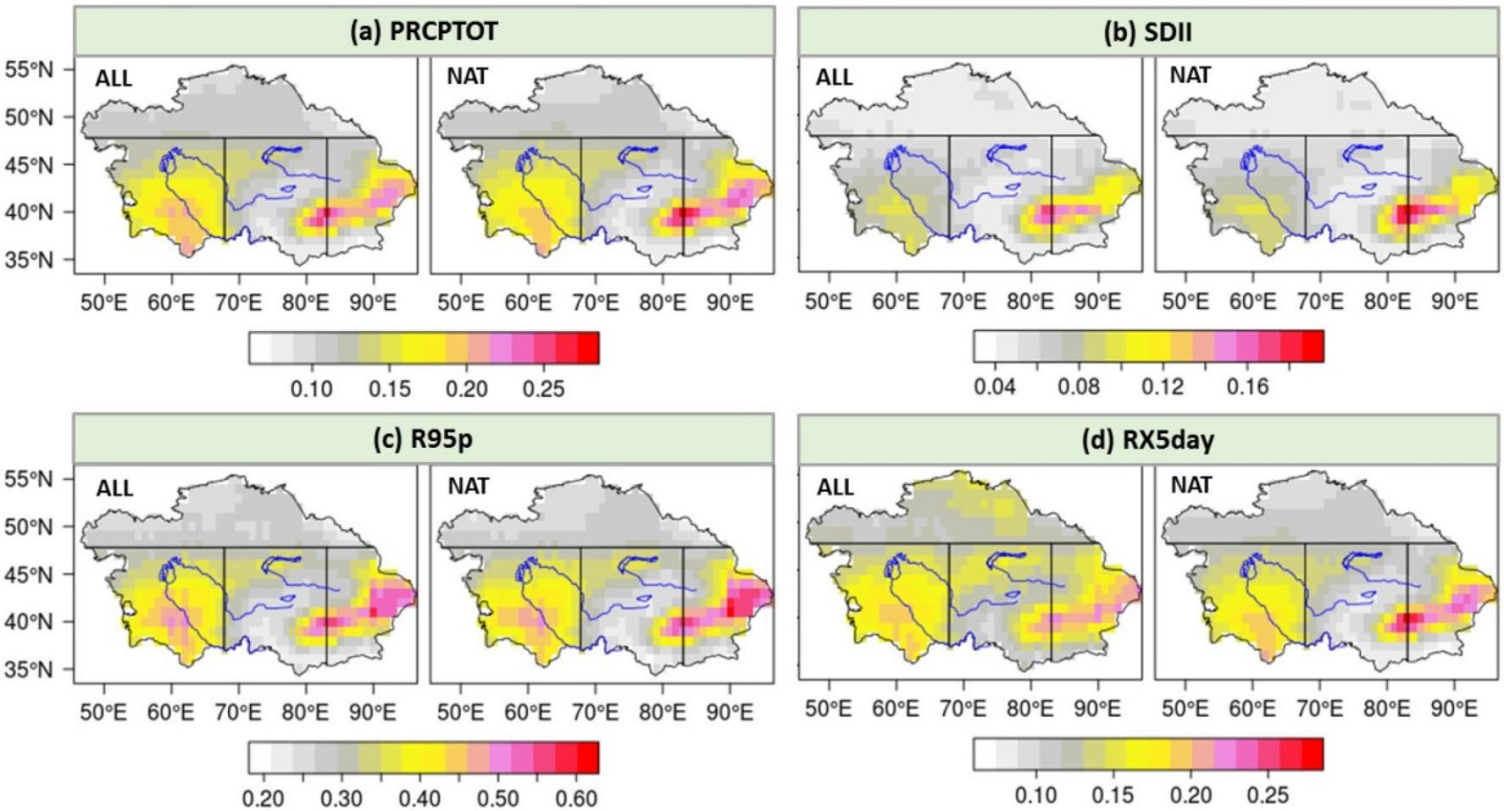
Spatial distribution of the mean multi-model ensemble (CMIP5 ALL and NAT simulations) Gini-coefficients for the (**a**) PRCPTOT, (**b**) SDII, (**c**) R95p and (**d**) RX5day in CA from 1961 to 2005. The subfigures were done in the software R 4.0.2 (https://cran.r-project.org/bin/windows/), and then the subfigures were merged by using the Microsoft PowerPoint 2013 software (https://www.microsoft.com/).

Figure 3 and Table 1 demonstrate the spatial distribution of the multi-model ensemble means of Gini-coefficients of the extreme precipitation indices for the CMIP5 NAT to ALL scenario from 1961 to 2005, suggesting a similar spatial distribution for each extreme precipitation index in the NAT and ALL scenario in CA. Among these 4 indices, the R95p has the highest mean Gini-coefficient value, exceeding 0.60 in the southern region of CA, while the SDII has the lowest mean Gini-coefficient, less than 0.08 for most regions. Figure 3 also clearly illustrates that the WCA and ECA have higher mean Gini-coefficient values than the NCA and CCA for all 4 indices under both scenarios, revealing a relative higher temporal precipitation variability for all 4 precipitation indices in WCA and ECA.

**Table 1:** Mean Gini values in 4 sub-regions and the whole of CA for the historical simulations.

Indices	Scenario	NCA	WCA	CCA	ECA	CA
PRCPTOT	ALL	0.1078	0.1551	0.1175	0.1467	0.1306
	NAT	0.1063	0.1524	0.1156	0.1524	0.1302
SDII	ALL	0.0474	0.0722	0.0618	0.0813	0.0650
	NAT	0.0485	0.0713	0.0621	0.0846	0.0655
R95P	ALL	0.2657	0.3801	0.2986	0.3774	0.3284
	NAT	0.2634	0.3773	0.2979	0.3918	0.3301
RX5day	ALL	0.1250	0.1595	0.1345	0.1640	0.1447
	NAT	0.1242	0.1584	0.1337	0.1697	0.1455

**Figure 4:**
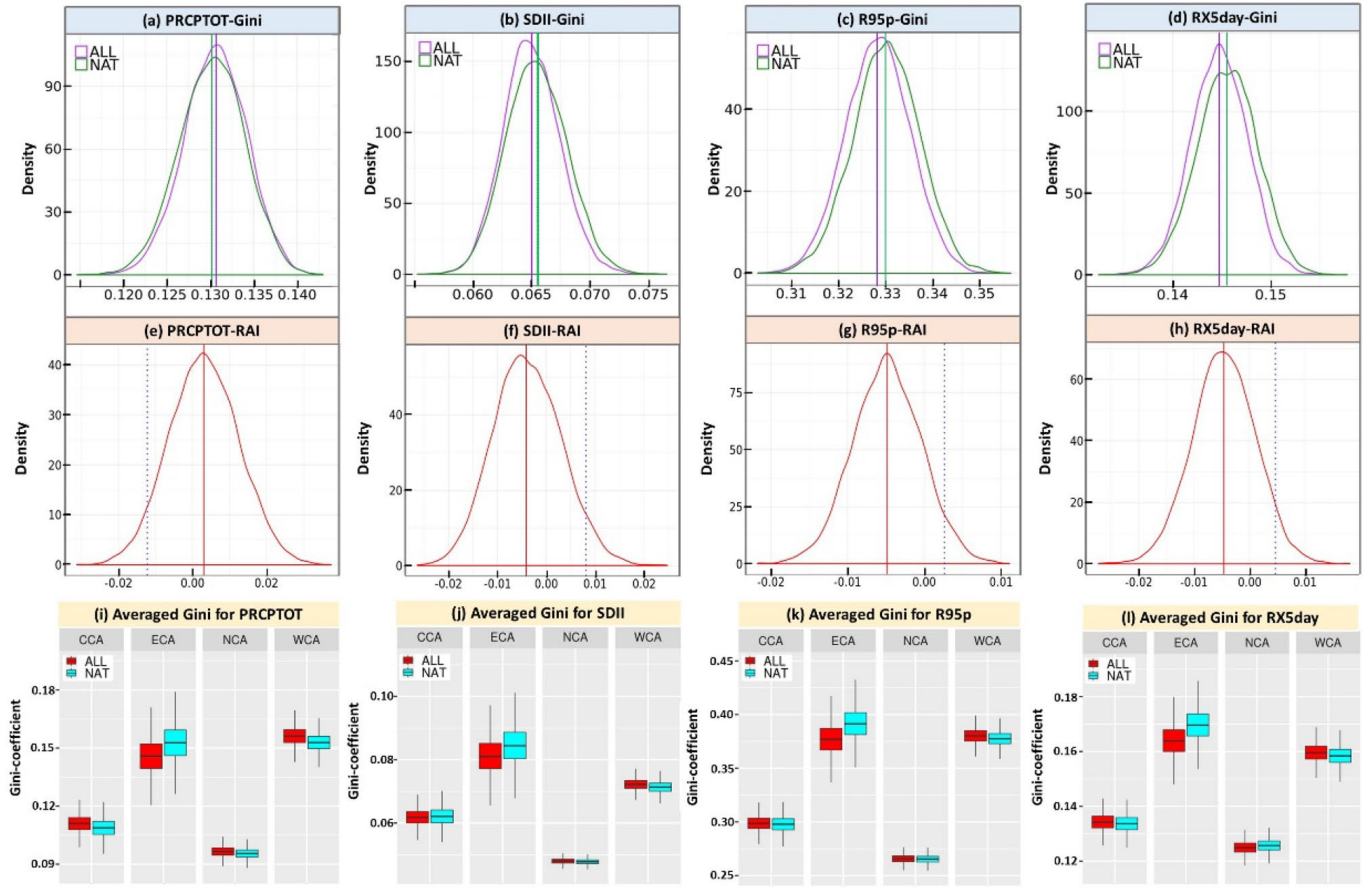
The probability distribution of the resampled averaged Gini coefficients for the (**a**) PRCPTOT, (**b**) SDII, (**c**) R95p and (**d**) RX5day, the distribution of the RAI values for the (**e**) PRCPTOT, (**f**) SDII, (**g**) R95p and (**h**) RX5day, and the boxplots of the resampled averaged Gini-coefficients for the (**i**) PRCPTOT, (**j**) SDII, (**k**) R95p and (**l**) RX5day regarding 4 sub-regions in CA, CMIP5 ALL and NAT scenario obtained by the bootstrapping procedure. The dash- lines in figures (**a**–**d**) represent the median (best estimate) value of the Gini-coefficients. The solid lines in figures (**e**–**h**) demonstrate the median (best estimate) value of the RAI and the dash- line indicates the 95th percentile value. The subfigures were done in the software R 4.0.2 (https://cran.r-project.org/bin/windows/), and then the subfigures were merged by using the Microsoft PowerPoint 2013 software (https://www.microsoft.com/).

The probability distribution of the averaged bootstrap resampled Gini-coefficients has been calculated from the multi-model ensemble means in CA, which is shown in Fig. 4a–d. A positive shift was noticed for the PRCPTOT from the CMIP5 NAT to ALL scenario and the value of the median Gini-coefficient increased from 0.1302 to 0.1306. On the contrary, a negative shift has been detected for the other 3 indices from the CMIP5 NAT to ALL scenario and the value of the median Gini-coefficient fell from 0.0655 to 0.0650 for the SDII, 0.3301 to 0.3284 for the R95p and 0.1455 to 0.1447 for the RX5day.

In Fig. 4e–h, we have plotted the probability distribution median and 95th percentile values of the Relative Anthropogenic Index (*RAI*) obtained by the bootstrapping procedure (as described in Method Section). In this study, the median *RAI* value (derived from the bootstrapped distribution) is determined as the best estimate, which is used to evaluate positive or negative changes. The PRCPTOP is slightly larger than 0 for the entire CA and the rest of the indices (including the SDII, R95p and RX5day) are less than 0 but did not pass the significance test. There is a RAI increase visible for the PRCPTOT (0.0030), suggesting a much higher temporal variability due to anthropogenic contribution in CA, while a *RAI* decrease for the SDII (− 0.0049), R95p (− 0.0041) and RX5day (− 0.0046) reveals less temporal variability due to anthropogenic contribution . Meanwhile, Fig. 4e clearly shows that the 5th percentile *RAI* values of the PRCPTOT (see dash-line) amounts to less than zero, which means that the increase is not statistically significant in non-uniformity regarding the PRCPTOT (0.30%), due to anthropogenic forcing. On the contrary, Fig. 4f–h denote that the 95th percentile values of the *RAI* (see dash-line) of the SDII, R95p and RX5day are greater than zero, which reveals that the decrease is not statistically significant in non-uniformity of the SDII, R95p and RX5day by 0.49%, 0.41% and 0.46%, respectively due to anthropogenic forcing.

Based on the resampled averaged Gini-coefficients, the boxplots were drawn (Fig. 4i–l) for each precipitation index in 4 CA sub-regions under the CMIP5 ALL and NAT scenario. Except for the ECA region, the median averaged Gini-coefficient of the CMIP5 ALL scenario was generally greater than the value of the CMIP5 NAT scenario for all 4 precipitation indices, which is in line with the results of CA, indicating the human contribution to potential changes in temporal precipitation variability in CCA, NCA and WCA. Of them, WCA has the highest difference in median averaged Gini-coefficients between the CMIP5 ALL and NAT scenario (Table 1), which means that the largest increase in non-uniformity of 4 precipitation indices was noticed in WCA (due to anthropogenic forcing). Among 4 sub-regions, NCA has the lowest median averaged Gini-coefficient values, illustrating the lowest temporal precipitation variability for the ALL and NAT scenario in northern CA. Also, regardless of the CMIP5 ALL or NAT scenario, the resampled averaged Gini-coefficients of WCA and ECA are higher than those of NCA and CCA. The possible reason for this phenomenon might be explained by the fact that there is a bigger precipitation frequency and larger precipitation amounts (and rainy months) are also noticed in NCA and CCA than in WCA and ECA, reflecting the higher magnitude of temporal variability in historical simulations in WCA and ECA. Figure 4i–l also clearly denotes that, in ECA, the NAT simulations have higher median Gini-coefficients (than those of the ALL simulations) for 4 sub-regions, indicating that the temporal variability of the annual precipitation is likely to decrease due to anthropogenic forcing in ECA.

**Figure 5:**
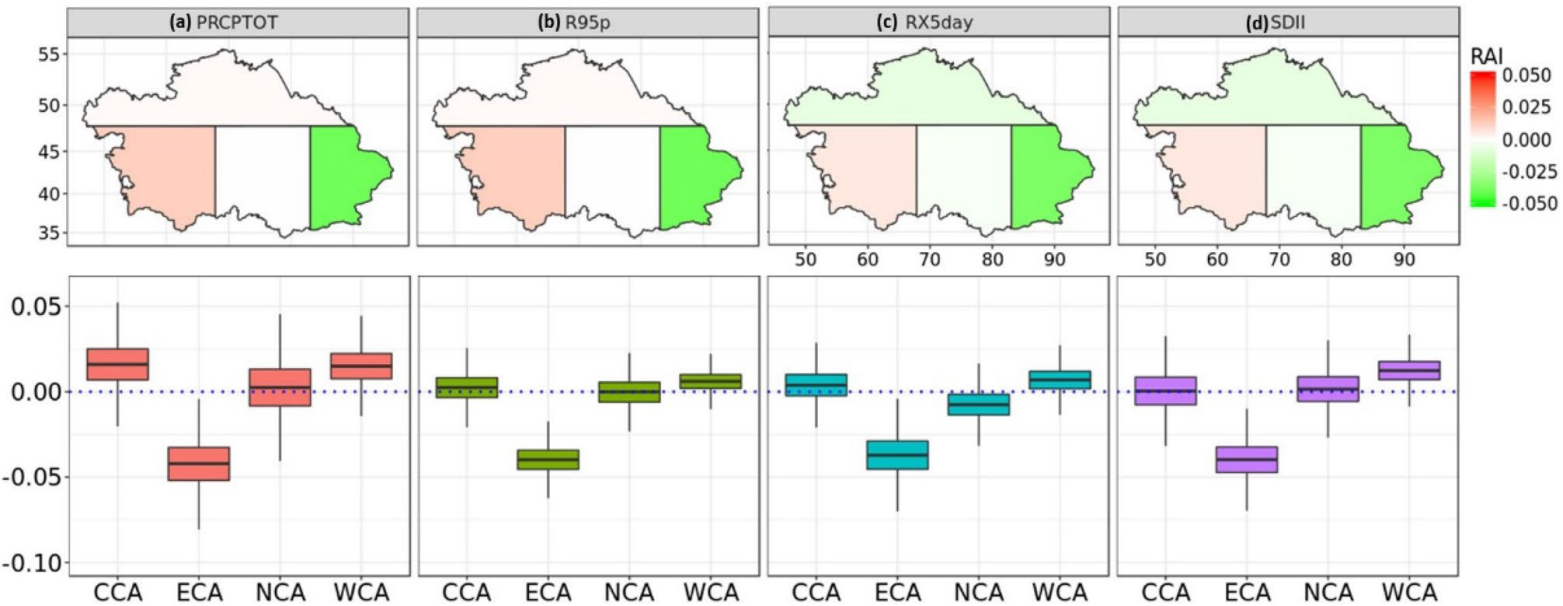
The figure on the upper panel shows the spatial distribution of the best temporal RAI estimates for the (**a**) PRCPTOT, (**b**) SDII, (**c**) R95p and (**d**) RX5day for 4 sub-regions in CA. The bottom panel indicates the corresponding uncertainty range (5th to 95th percentile) of the temporal RAI estimates for 4 sub-regions in CA. The horizontal lines in the box demonstrate the best estimate (i.e. median value). The subfigures were done in the software R 4.0.2 (https://cran.r-project.org/bin/windows/), and then the subfigures were merged by using the Microsoft PowerPoint 2013 software (https://www.microsoft.com/).

Figure 5 represents the best RAI estimates of the extreme precipitation indices in 4 sub-regions of CA, along with their uncertainty range at 95% and 5% limits (maximum and minimum values). From the figure, we could conclude that the best RAI estimates in CCA and WCA are generally greater than zero, which suggests that anthropogenic forcing in these 2 regions caused more unbalanced extreme precipitation indices from 1961 to 2005. However, the effects did not pass the significance test. Figure 5 also shows an outstanding feature, namely that the ECA region exhibits the lowest anthropogenic influence on the variability of the PRCPTOT, SDII, R95p and RX5day, the best *RAI* amount to -0.0422, -0.0398, -0.0397 and -0.0373, respectively. Meanwhile, according to the value of the 5% limit, there is a statistically significant (95% confidence) decrease in the non-uniformity of the PRCPTOT, SDII, R95p and RX5day by 4.22%, 3.98%, 3.73% and 3.97%, respectively. This outcome indicates that a statistically significant decrease in uniformity of the PRCPTOT, SDII, R95p and RX5day by 4.22%, 3.98%, 3.73% and 3.97% was detected in the ECA area from 1961 to 2005 (due to anthropogenic forcing). Hence, some CA regions with significant human activities induced changes in the temporal distribution. Most regions of CA demonstrate however insignificant human activities (induced non-uniformity).

## Discussion

Based on the CMIP5 ALL and NAT simulations, we have evaluated the impact from anthropogenic forcing on the trend and non-uniformity of the precipitation amounts (PRCPTOT), intensity (SDII) and extremes (R95p and RX5day) in CA. The CMIP5 ALL simulations overestimated the Gini-coefficients in the southwestern part of CA and the centre of the Tarim River Basin, where only a few meteorological stations are located. The results might reflect that the current GCM simulations show the general weakness of the daily variation of the precipitation prediction in these regions with sparse gauged data^34,35^. Overall, the CMIP5 ALL simulations could generally capture the spatial pattern of the temporal variations, which prove to be consistent with previous studies.

Compared with previous studies^26,36^, the whole of CA suffers from more significant changes in the precipitation extreme events. For example, an augmenting tendency was detected for most precipitation indices (including the PRCPTOT, SDII, R95p and RX5day), causing wetter conditions in CA from 1938 to 2005^37^. Another instance of most precipitation indices shows the spatial diversity and heterogeneity and generally the increasing trend of the PRCPTOT, SDII, R95p and RX5day which was substantial in the Tianshan Mountain and hill regions^38^ but not obvious in the midland desert and depression areas^37^. All these findings comply with the trend variability of the precipitation analyzed in this study, clearly suggesting that anthropogenic forcing has risen the likelihood of heavy precipitation events in CA, especially in the alpine areas.

Among 4 sub-regions, the ECA region was the only region with a statistically substantial rise in non-uniformity of the PRCPTOT, SDII, R95p and RX5day by 4.22%, 3.98%, 3.73% and 3.97%, respectively from 1961 to 2005 due to anthropogenic forcing. The results reveal an increase in the temporal equality of the extreme precipitation indices in northwest China, which is consistent with the results analyzed by Sun et al.^39^. A possible factor (causing this result) might be the low value of the precipitation frequency and amount in these regions with a mean annual precipitation of less than 200 mm^40^. Although the extreme precipitation indices exhibit a rising tendency with an increase of the precipitation amounts from 1961 to 2005^41^, a rise within a certain range will probably trigger a drop in non-uniformity (i.e. decrease in unevenness) of the precipitation indices.

Several studies have explored the potential linkage between the precipitation extremes and the large-scale atmospheric circulation in CA, suggesting that the large-scale atmospheric circulation changes play a vital role in the changes of the precipitation amounts and extreme precipitation events^32,42–45^. The large-scale atmospheric circulation is controlled by a series of large-scale climate indices, including the Atlantic Multidecadal Oscillation (AMO), North Atlantic Oscillation (NAO), Arctic Oscillation (AO), Pacific Decadal Oscillation (PDO) and El Nino-Southern Oscillation (ENSO). All these indices have strong effects on the differences in the moisture transport pathways, eventually affecting the precipitation patterns and extremes^36,46,47^. However, the influence of the large-scale atmospheric circulation is rather complex^42,43^ and further work is still needed in order to quantify the internal regimes more precisely.

Overall, based on the changes of the PRCPTOT, SDII, R95p and RX5day from 1961 to 2005, this study has assessed the impact from anthropogenic forcing to the trend and non-uniformity on the extreme precipitation events in CA. The main results and conclusions could be summarized as follows: (1) overall, the CMIP5 ALL simulations demonstrated a little overestimation for the Gini-coefficients but reasonably characterized the spatial pattern; (2) there is a clear signal that the radiative forcing changes (mainly driven by human activities) have significantly increased the extreme climate indices in CA and the median trend for the PRCPTOT, SDII, R95p and RX5day rose from 0.59 to 2.19 mm/decade, 0.004 to 0.019 mm/decade, 0.25 to 1.39 mm/decade and 0.07 to 0.21 mm/decade from 1961 to 2005, respectively; and (3) there is a statistically insignificant decrease in non-uniformity of the PRCPTOT, SDII and RX5day in CCA and WCA regions, while a statistically significant (95% confidence) increase in non-uniformity in the ECA region, reflecting the difference in various regions regarding the impact from anthropogenic forcing on non-uniformity on the extreme precipitation events. Due to the simple ecological structure, fragile ecosystem and weak stability in CA, the changes in the precipitation extremes probably cause a series of ecological, environmental and social sequences. The outcomes obtained from this study could help so as to fully understand the role of anthropogenic forcing in the precipitation extremes’ changes and might finally contribute to the development of water resource management strategies for protecting the fragile ecosystem.

## Data and method

### Observed dataset

Overall, the meteorological observational network was established during the Soviet era and the network is still unsatisfactory in CA. After the fall of the Soviet Union, there has been a persistent downward trend in the quantity and quality of the measurements at most meteorological stations. Many meteorological stations were closed due to a lack of funds, causing incomplete long-term datasets at most stations^26,36^. In this study, the HadEX3 dataset was selected as the observed dataset; this dataset was chosen because it is the latest global dataset of the land surface extreme climate indices derived from the daily station data. We have checked and confirmed that it generally covers the existing meteorological stations with long-term data in CA. The dataset was first calculated at each station and then interpolated onto a global grid over land with a 1.875° longitude × 1.25° latitude spatial resolution^48^, which has been successfully used to evaluate the detailed changes in the regional climate and to validate the climate model simulations^49,50^.

### GCM datasets

We used 2 simulation sets, including the historical experiment and the historical Nat experiment from 15 GCMs (Table S2) downloaded from the CMIP5 multi-model data archive. In general, the historical experiment has been forced by the observed atmospheric composition changes (reflecting the anthropogenic and natural sources) and the time-evolving land cover^51^. While the historical Nat experiment was only run with time-dependent natural forcing (solar and volcanic aerosol), hence providing estimates of the Earth’s climate without the anthropogenic influences^51^. In order to obtain a better comparison with the observed data, the time period of the historical and historicalNat simulations was selected from 1961 to 2005. All precipitation extreme indices from the CMIP5 simulations were downloaded online (http://www.cccma.ec.gc.ca/data/climdex/index.shtml, accessed in 2020, July).

### Extreme precipitation indices and data processing

In order to describe the climatic extremes’ changes, totally 27 climate indices were defined and developed by the Expert Team on Climate Change Detection and Indices (ETCCDI, see http://www.climdex.org/indices.html)14, which have been widely applied to characterize and evaluate the evolution of the climate and weather extremes^45,52^. In this study, 4 indices, including the total annual wet-day precipitation (PRCPTOT), the maximum 5-day precipitation amount (RX5day), the simple daily intensity index (SDII) and the extremely wet days (R95p) have been selected so as to evaluate the spatial–temporal variability of the extreme precipitation events (Table S3). Of them, the PRCPTOT could be utilized to assess the changes in total precipitation; the SDII represents the precipitation intensity; the R95p is a percentile-based threshold index to evaluate the precipitation events during very wet days and the RX5day demonstrates an absolute index, which is usually applied to describe changes in potential flood risks (as heavy rain conditions over several consecutive days which could contribute to flooding)^53^. In order to unify the resolution of different datasets, we re-gridded all extreme precipitation indices to a common grid of a 1° latitude × 1° longitude using a remapping procedure^54^. Meanwhile, according to the sub-regions including the NCA, WCA, CCA and ECA from Figure S1, we have carried out a regional analysis to fully evaluate the changes in the extreme precipitation events.

### Trend estimation and testing methods

The linear least-squares’ regression method^55^ was used to assess the trend of the extreme precipitation indices on the grid and regional scales (NCA, WCA, CCA and ECA) for CA. The statistical significance for each indice has been checked by using the Mann–Kendall trend test, which is a non-parametric method to effectively evaluate the trend of extreme climatic events^56^. The trends were obtained from the arithmetic mean values of the annual extreme precipitation indices, respectively for the ALL and NAT scenario.

### Gini coefficient

The Gini coefficient has been applied as a measure for the income inequality in a society^57^ but more recently this has been done with a quantification uniformity in the time series of the climate variables^58,59^, calculated by the following equation:

1 $$G = \frac{1}{n}\left( {n + 1 - 2*\left( {\frac{{\mathop \sum \nolimits_{i = 1}^{n} \left( {n + 1 - i} \right)*y_{i} }}{{\mathop \sum \nolimits_{i = 1}^{n} y_{i} }}} \right)} \right),$$

where *n* indicates the total number of years and $y_{i}$ illustrates the PRCPTOT (or SDII, R95P, RX5day) in a particular year *i*. The Gini index ranges from 0 to 1, with 1 associated to the maximal disparity and 0 denoting a complete uniformity. Therefore, the Gini coefficient with small values suggests more uniformity in the temporal variation of 4 extreme precipitation indices, while the Gini coefficient with higher values indicates a higher non-uniformity in the temporal variation of the precipitation indices. The diagram of the Gini coefficient is presented in Figure S3. The Gini coefficient is easy to interpret in different geographical environments, other measures for the variability methods, such as the standard deviation, are a possibility distribution of the data and prove to be scale-sensitive^60^.

In this study, we compared the Gini coefficient of the extreme precipitation indices under the CMIP5 ALL and NAT scenario to confirm whether there is a disparity or identity of the temporal variability in CA. In order to compute the regional Gini coefficient, we have considered the average value of the gridded Gini-coefficients in 4 sub-regions and finally, we noticed that each region has a single value of the Gini-coefficient for each CMIP5 historical and historicalNat scenario. Afterwards, we employed the Bootstrapping resampling procedure to randomly select GCM models with a repetition from the pool of 42 CMIP5 ALL or NAT simulations to calculate their ensemble means. In this study, we obtained 10,000 spatially averaged Gini coefficients, which could be used to represent the inter-model variability. In order to understand the impact from anthropogenic forcing on the extreme precipitation events in different sub-regions, we calculated the probability distribution functions (PDFs) for the ALL and NAT simulations of each sub-region.

### Attribution analysis

In order to assess the extent of the anthropogenic or natural influences for climate change or extreme events, scientists have developed climate models to simulate the changes in precipitation with and without anthropogenic influences by setting various scenarios^61^. More recently, this typically involved estimates of the fractional attributable risk (*FAR*), a common technique to quantify the attributable risk of extreme precipitation events in the model analysis. The uncertainty of FAR has generally been estimated for its statistical significance by means of the bootstrapping method. Based on the Gini coefficient, we similarly used the Relative Anthropogenic Index (*RAI*) developed by Konapala et al.^62^ so as to quantify the effects of human activities on the temporal precipitation variability. The *RAI* has been calculated in the following equation^62^:

2 $$RAI = \frac{{G_{ALL} - G_{NAT} }}{{G_{ALL} }},$$

where $G_{NAT}$ and $G_{ALL}$ indicate the Gini coefficient of the CMIP5 NAT and ALL simulation. A negative *RAI* suggests that human activities lead to an increase in uniformity in the time series of the extreme precipitation indices, while a positive *RAI* value indicates rather the opposite. In the context of resource management, a positive *RAI* value indicates that the challenges faced in water resource and ecosystem management have been increasing, while a negative *RAI* value means that the variability was reduced, so the water resources are easier to manage as a result.

In order to evaluate the *RAI* uncertainty, the Bootstrapping resampling procedure has been applied to generate 10,000 sub-samples from 42 CMIP5 realizations and to rank these so as to extract the 5th and 95th percentile *RAI* values. Thus, the distribution of 10,000 *RAI* values could reflect the uncertainty associated with the use of different models and provides a basis to communicate the *RAI* ranges. A positive median of the *RAI* is statistically significant if its 5th percentile value is also positive, while a negative *RAI* median is statistically significant if its 95th percentile value is also negative. Finally, the *RAI* estimates could be applied to reflect the influence of anthropogenic forcing on the precipitation non-uniformity, along with its significance.

Figure S1 was done using the software ArcGIS 10.5.1 software (https://desktop.arcgis.com), and Fig. S3 was generated with the Microsoft Visio 2013 software (https://www.microsoft.com/). Panels for Figs. 1, 2, 3, 4, 5, and S2 were done in the software R 4.0.2 (https://cran.r-project.org/bin/windows/). We merged the panels of the different figures using the Microsoft PowerPoint 2013 software (https://www.microsoft.com/).

## Supplementary Information﻿

Supplementary Information.
