# Supplementary material for: Attribution of changes in the trend and temporal non-uniformity of extreme precipitation events in Central Asia
Source: Sci Rep. 2021 Jul 22;11:15032. doi: 10.1038/s41598-021-94486-w (PMC8298420; doi:10.1038/s41598-021-94486-w)
Supplement: Supplementary file 1 — Supplementary Information. [file 41598_2021_94486_MOESM1_ESM.docx]

# Supplementary material

# Attribution of changes in the trend and temporal non-uniformity of extreme precipitation events in Central Asia

**Shan Zou^1, 2, 3, 4, 5, 6, 7^,** **Jilili Abuduwaili^1, 2, 3^, Weili Duan^1, 3, *^, Jianli Ding^4^, Philippe De Maeyer^1, 3, 5, 6, 7^ , Tim Van De Voorde ^5, 6, 7^ and Long Ma^1, 2, 3^**

^1^ State Key Laboratory of Desert and Oasis Ecology, Xinjiang Institute of Ecology and Geography, Chinese Academy of Sciences, Urumqi 830011, China;

^2^ Research Centre for Ecology and Environment of Central Asia, Chinese Academy of Sciences, Urumqi 830011, China

^3^ University of Chinese Academy of Sciences, Beijing 100049, China

^4^ College of Resource and Environment Sciences, Xinjiang University, Urumqi 830046, China

^5^ Department of Geography, Ghent University, 9000 Ghent, Belgium

^6^ Sino-Belgian Joint Laboratory of Geo-Information, 9000 Ghent, Belgium

^7^ Sino-Belgian Joint Laboratory of Geo-Information, Urumqi 830011, China

*Corresponding author: duanweili@ms.xjb.ac.cn (Weili Duan)

**Table S1.** Coordinates of the four sub-regions and the whole of Central Asia (CA) used in this study

| **Name** | **Abbreviation** | **Coordinates** |
| --- | --- | --- |
| Northern CA | NCA | 48–56°N, 46–97°E |
| Western CA | WCA | 34–48°N, 46–68°E |
| Central CA | CCA | 34–48°N, 68–83°E |
| Eastern CA | ECA | 34–48°N, 83–97°E |
| Whole CA | CA | 34–56°N, 46–97°E |

**Table S2. GCM models and their corresponding realizations.**

| Number | Model name | Realizations | Resolution (lat × lon) |
| --- | --- | --- | --- |
| 1 | BCC-CSM1-1 | r1i1p1 | -2.8°× 2.8° |
| 2 | BNU-ESM | r1i1p1 | -2.8°× 2.8° |
| 3 | CanESM2 | r1i1p1, r2i1p1, r3i1p1, r4i1p1, r5i1p1 | -2.8°× 2.8° |
| 4 | CCSM4 | r1i1p1, r2i1p1, r4i1p1, r6i1p1 | -1.25°× 1° |
| 5 | CNRM-CM5 | r1i1p1, r2i1p1, r3i1p1, r4i1p1, r5i1p1, r8i1p1 | -1.4°× 1.4° |
| 6 | CSIRO-Mk3-6-0 | r1i1p1, r2i1p1, r3i1p1, r4i1p1, r5i1p1 | -1.87°× 1.85° |
| 7 | GFDL-CM3 | r1i1p1, r3i1p1, r5i1p1 | 2.5°× 2° |
| 8 | GFDL-ESM2M | r1i1p1 | -2.5°× 2° |
| 9 | HadGEM2-ES | r1i1p1, r2i1p1, r3i1p1, r4i1p1 | 1.875 × 1.25° |
| 10 | IPSL-CM5A-LR | r1i1p1, r2i1p1, r3i1p1 | -3.75°× 1.9° |
| 11 | IPSL-CM5A-MR | r1i1p1, r2i1p1, r3i1p1 | -2.5°× 1.3° |
| 12 | MIROC-ESM | r1i1p1, r2i1p1, r3i1p1 | -2.8°× 2.8° |
| 13 | MIROC-ESM-CHEM | r1i1p1 | -2.8°× 2.8° |
| 14 | MRI-CGCM3 | r1i1p1 | -1.1°× 1.1° |
| 15 | NorESM1-M | r1i1p1 | -2.5°× 1.9° |

**Table S3**. Definition and description of 4 precipitation indices

| **ID** | **Type** | **Indicator name** | **Definition** | **Unit** |
| --- | --- | --- | --- | --- |
| PRCPTOT | Absolute index | Annual total wet-day precipitation | Annual total PRCP in wet days (RR>=1mm) | mm |
| SDII | Absolute index | Simple daily intensity index | Annual total precipitation divided by the number of wet days (defined as PRCP>=1.0mm) in the year- | mm/day |
| R95p | Percentile-based threshold  index | Extremely wet days | Annual total PRCP when RR>95^th^ percentile of precipitation on wet days | mm |
| RX5day | Absolute index | Max 5-day precipitation amount | Yearly maximum 5-day precipitation | mm |

The abbreviations are as follows: RR, daily precipitation. A wet day is defined when RR≧1 mm and a dry day when RR<1 mm. The temperature indices are included for the completeness but some have not been analyzed further (in this article).

**
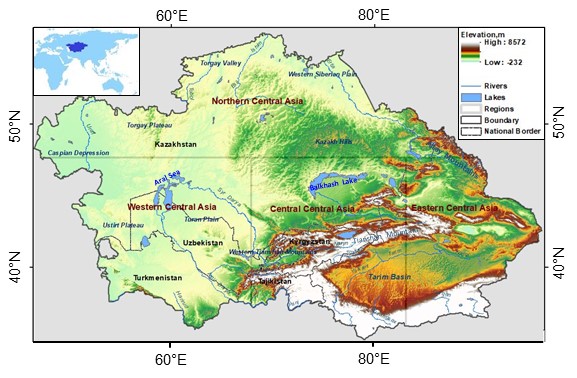
**

**Figure S1.** Location of the study area (Map Review (Inspection) Number: GS [2019]3266). The map was generated with the ArcGIS Desktop 10.5.1 software (https://desktop.arcgis.com).


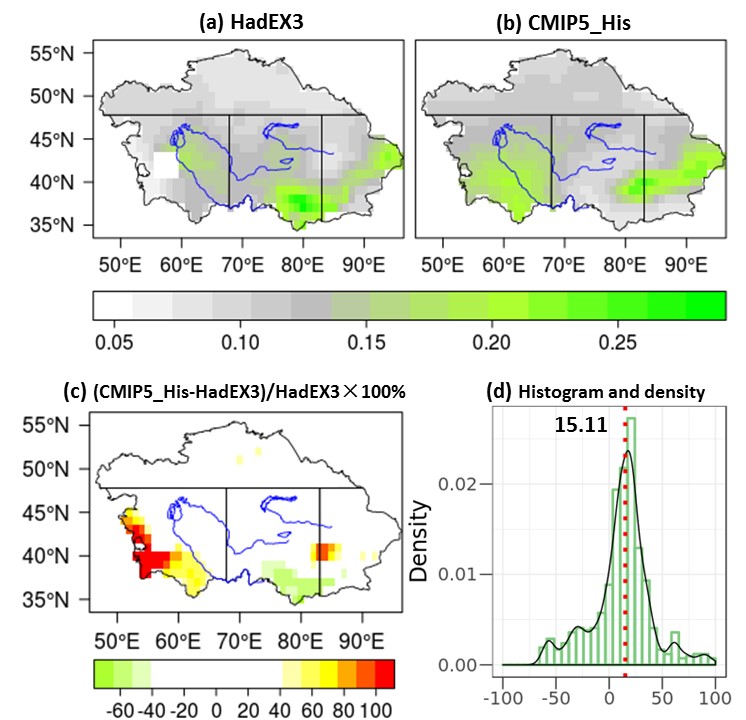


**Figure S2.** Comparison of the Gini-coefficients for the PRCPTOT (mm/year) between the observed (HadEX3) value and the mean multi-model ensemble (CMIP5 ALL) from 1961 to 2005. (a) Spatial distribution of the HadEX3 Gini-coefficients, (b) Spatial distribution of the CMIP5 ALL Gini-coefficients, (c) Spatial distribution [(CMIP5- HadEX3)/HadEX3] of the Gini-coefficients’ bias (the unit is %) and (d) Histogram and density plots [(CMIP5- HadEX3)/HadEX3] of the Gini-coefficients’ bias (the unit is %). The red dash-dot line represents the average value for all grids. The subfigures were done in the software R 4.0.2 (https://cran.r-project.org/bin/windows/), and then the subfigures were merged by using the Microsoft PowerPoint 2013 software (https://www.microsoft.com/).


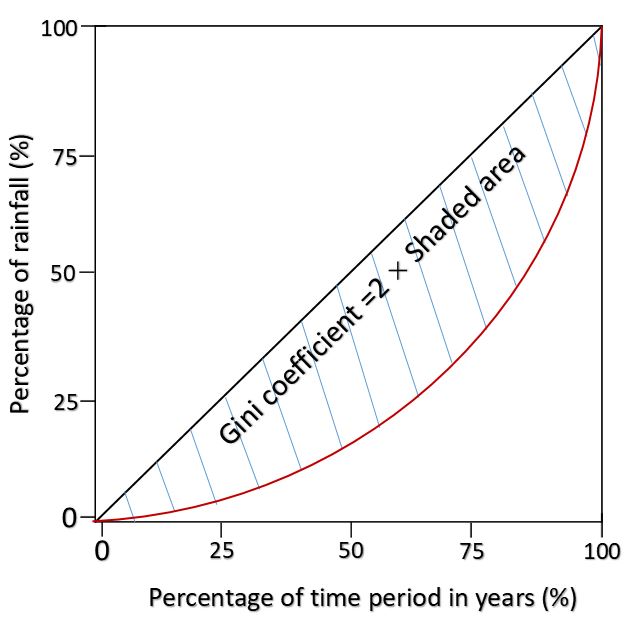


**Figure S3.** Graphical representation of the Gini coefﬁcient for the temporal variability: the PRCPTOT has been accumulated first after ranking and converting into percentiles (as shown by the red curve). Then, the Gini value was estimated as twice the area between the absolute equality line (1:1line) and the ranked distribution, depicted by the slash shaded area. Hence, the larger the slash area, the more variability. The figure was generated with the Microsoft Visio 2013 software (https://www.microsoft.com).
